# Environmental and safety risk assessment for sustainable circular production: Case study in plastic processing for fashion products

**DOI:** 10.1016/j.heliyon.2023.e21352

**Published:** 2023-10-20

**Authors:** Anna Mazzi

**Affiliations:** University of Padova, Department of Industrial Engineering, SAM Lab, Padova, Italy

**Keywords:** Environmental impacts, Occupational health and safety risks, Integrated risk management, Industrial waste minimization, Italian case study

## Abstract

Even if sustainability and circularity are the most challenging goals today, industrial waste minimization is rarely discussed and practical methods to reduce risks related to hazardous waste in manufacturing processes are not frequently applied yet. The case of Italian company specialized in plastic processing for fashion products, has the chance to design and test a new integrated methodology to reduce the risks for ecosystem and workers associated to hazardous waste. Focusing the attention to standard operations, extraordinary conditions, and emergency situations, all activities included in waste collection, storage and transport are identified and the risks associated to the environmental impacts and the occupational health&safety are analysed. Research results demonstrate the opportunity to adopt one method to analyse both environmental and health&safety risks associated to activities and conditions involved in industrial waste management. The case study confirms the relevance of integrated approaches and the necessity of simplified tools to support companies in adopting integrated risk management.

## Highlights

• Risk minimization in industrial waste production for sustainable and circular plastic processing.
• Integrated risk assessment to minimize environmental and health&safety impacts.
• Focus on standard operations, extraordinary situations, and emergency conditions.
• Easy-to-use tool for integrated EHS management in operations.

## 1 Introduction

Sustainable production and circular economy represent the main ambitious targets worldwide: the 12th Sustainable Development Goal (SDG) of the United Nations program, focused on “Responsible Consumption and Production”, involves all economic sectors to contribute to the “2030 Agenda” with technical and technological solutions that reduce negative impacts related to industrial processes [1]. The circular economy business model, focused on the “closing the loop” perspective, intends to solve at the same time the two main problems of the current economic model: increasing amount of waste and decreasing availability of resources [[2], [3], [4]]. To obtain consistent results in terms of sustainability and circularity, companies are encouraged to substantially reduce waste generation primarily through prevention measures, secondly through reuse solutions, and finally through recycling options [5,6]. In the last decade, for scientists, practitioners and policy-makers the relevance of Sustainable-Circular Production (SCP) increases [7,8] and now the 3R (Reduce-Reuse-Recycle) framework is the undisputed reference in waste&resource management [9,10]. However, several obstacles still inhibit implementation of SCP practices in companies, and the challenge of the 3R transition remains formidable [11]. Notable reviews contribute to analyse the fragmented knowledge about the concepts of circular production [12,13], as well as several authors underline both internal and external barriers to sustainable production model (e.g. Refs. [[14], [15], [16]]).

At experience level, huge amount of papers focuses the attention on recycling technologies and recycling/recycled materials, however rarely the efforts of scientists are related to reduce industrial waste production [[17], [18], [19]]. Otherwise, to move industries to the new SCP model, recycling is not the unique solution: preventive measures are needed to minimize industrial waste and resource consumption, by rethinking of manufacturing processes, eliminating hazardous substances, minimizing waste generation, optimizing resource use and reuse, and improving performance of scrap management (e.g. Refs. [[20], [21], [22], [23]]). At firm level, the interest in methods and tools to improve circularity and sustainability of processes is growing among managers [24,25]. Otherwise, while the theoretical discussion on circular business models is abundant, practical uptake remains often vague and limited, and SCP is still very complex to implement by companies (e.g. Refs. [[26], [27], [28]]). Focused on concrete business targets, enterprises need easy-to-use tools to apply the SCP model within operations management (e.g. Refs. [[29], [30], [31]]).

On the other hand, when it comes to sustainability, the environmental dimension is not the only one that needs to be considered: Triple Bottom Line (TBL) is the comprehensive perspective of corporate sustainable business model, in which simultaneously economic, social, and environmental benefits are pursued [32,33]. More and more companies have already included TBL in their mission, compelled by the increasing consciousness of consumers and market [[34], [35], [36]]. Recently a plethora of papers has been published, mainly focused on environmental and economic benefits related to material and resource recirculation, otherwise rarely scientific literature is dedicated to the social concerns of circularity [37,38]. In fact, while the environmental convenience of 3R is abundantly proved, the scientific debate concerning the social dimension of circularity is still in its infancy [[39], [40], [41]], and in particular safe&healthy working conditions of 3R options seem unexplored [[42], [43], [44]].

The research intends to enrich the scientific discussion concerning the SCP at firm level focusing the attention on the industrial hazardous waste minimization. Through the case study of a plastic processing Italian company, the research goal is to design and test new easy-to-use method to assess and reduce the environmental, health and safety (EHS) risks associated to waste production and collection.

The paper is structured as follows. In section 2, literature review is presented, to obtain scientific background and reference criteria to design integrated EHS risk assessment for industrial waste minimization. In section 3, coherently with the international guidelines, the new methodology is elaborated to identify, assess and evaluate EHS risks related to industrial waste management; moreover, formulas to quantify EHS risks are explained, and the case study of Italian manufacturing company is presented. In section 4, results from the application of EHS risk management methodology to the case study are presented and discussed. In section 5, conclusions about validity and replicability of experience are summarized and further research perspectives are underlined.

## 2 Background

Since the 1980s, both market's needs and legislative requirements have induced companies around the world to pay attention to EHS concerns [45,46].

Many scientists demonstrate the opportunity and convenience in adopting integrated tools to reduce environmental impacts related to production activities, while at the same time reducing the health&safety risks associated to processes (e.g. Refs. [[47], [48], [49]]). Multiple internal benefits derive from the integration of EHS risks for example, implementation of common requirements and lower internal costs, motivation of human resources, and organizational improvements [[50], [51], [52]]. Other advantages deriving from integrated EHS risks management include maximization of business management results [53] and synergic advantages in performance, consciousness and accountability [54,55]. However, enterprises generally struggle to adopt integrated EHS risk assessment methods [[56], [57], [58]]. Although prevention of environmental impacts and avoidance of health&safety damages have the same approach, they pursue different objectives, consider different issues, and therefore need different competences [59,60]: the environmental management points to quantify and reduce environmental impacts associated to processes and activities with a life cycle approach [61], otherwise, the health&safety management aims to identify, evaluate, and reduce risks for health&safety in working conditions, related to materials, components, machineries, and plants, with a probabilistic approach [62,63].

Instead, the key element characterizing both environmental and health&safety management is the systematic approach of risk management (RM) [64,65]: through the steps of identification, analysis and evaluation, RM allows organizations to predict danger factors for their activities and supports them in planning durable business [66,67].

Increasing interest about integrated RM is testified by several experiences, especially in energy, wastewater and building sectors (e.g. Refs. [[68], [69], [70]]). RM is also recently adopted to support strategic business management, especially in the cases of scarce resources and critical supply chains (e.g. Refs. [[71], [72], [73]]). Although RM is applicable to any kind of risk and can be robust reference to define strategies and manage operations [74,75], integrated RM is still not widespread among companies and not systematically used [76,77].

At industrial level, one of the most critical activities in terms of EHS risks is the industrial waste production and collection [[78], [79], [80]]. Detailed EHS management rules are essential for example in case of hazardous waste, to avoid severe consequences and minimize negative risks for both the environment and workers [[81], [82], [83]]. In manufacturing operations, activities related to collection and disposal of residues are characterised by risks due to the dangerousness of substances and materials, which can determine negative consequences for both the environment and the occupational health&safety (e.g. Refs. [[84], [85], [86], [87], [88],^89^]). Moreover, in waste management, particular attention should be paid not only to routine procedures but, above all, to dangerous situations that may arise from extraordinary conditions or emergency situations [90]. RM could be adopted for both standard and non-standard situations; otherwise, companies frequently adopt different methods to quantify risks associated to standard and extraordinary conditions, impeding a unitary risk evaluation [91,92].

According to SCP model, industrial waste minimization represents strategic target for companies today, with significant durable advantages [[93], [94], [95]]. International recommendations sustain prevention and precaution of EHS risks associated to waste management, to avoid negative environmental impacts [96] and achieve organizational and economic benefits [97,89].

Even if growing number of companies declares the commitment in sustainability and circularity, the importance of risk prevention in industrial waste is often overlooked. As denounced by several authors, rarely practitioners and managers are able to minimize EHS risks associated to waste and scraps, due to lack of specific skills and simple support tools (e.g. Refs. [[98], [99], [100], [101]]). At industrial level, the efforts for SCP are frequently devoted to discover new solutions to recycle materials rather than avoid waste production or reduce EHS risks associated to industrial waste (e.g. Refs. [[102], [103], [104]]). In particular hazardous waste minimization is rarely assumed as strategic business goal by companies, due to lack of competences among managers to implement concrete actions of SCP in operations [105,106]. Further efforts are needed to demonstrate the importance of waste minimization and risk prevention at business level, and easy-to-use tools are needed to guide companies to put into practice the SCP model [[107], [108], [109]]. Moreover, RM approach is successful to quantify negative impacts associated to waste production and collection into operations [[110], [111], [112]], as well as integrated assessment is needed to support managers to consider at the same time both environmental and health&safety parameters in operations management and in waste management [[113], [114], [115]].

From this overview, some key topics emerge to be further studied.

(i) Even if international community declares its intention to achieve SCP targets, effective tools to support companies in applying sustainable and circular practices are still limited.
(ii) Although the integration of EHS issues is essential to obtain effective sustainable practices, difficulties to implement it in operations remain among companies, due to the lack of easy-to-use tools.
(iii) Industrial waste is one of the most urgent areas in which risk minimization must be implemented, to improve processes related to collection and disposal of hazardous scraps, with prevention and precaution measures; nevertheless, in companies, the risks associated with waste management is still underestimated.
(iv) RM can solve problems associated to industrial waste management at company level, because it can be applied to reduce both environmental and health&safety criticalities associated to production, collection and delivery of hazardous materials and substances; nevertheless, its adoption by companies is uncommon.
(v) Scientists encourage implementation of integrated RM, to better understand and reduce EHS risks associated to operations; however, RM framework is rarely known by companies and poorly used to obtain SCP goals.
(vi) Concerning hazardous waste collection and disposal, extraordinary conditions and emergency situations require specific risk assessment to avoid severe consequences; nevertheless, risk assessment methods generally provide for ad-hoc criteria, then unitary evaluation in case of standard operations and extraordinary conditions is difficult.

To solve these emerging gaps, experimental research has been conducted, with the aim of design and test an easy-to-use tool for EHS RM to minimize risks of industrial waste in standard, extraordinary and emergency situations. To achieve the research goals, three complementary steps of research have been conducted:

a. To identify, assess and evaluate EHS risks of industrial waste management, ad-hoc methodology and formulas have been elaborated coherently with international references and guidelines, considering standards operations, extraordinary conditions and emergency situations.
b. To test the EHS RM methodology, an Italian company world leader in plastic materials processing has been considered, and detailed data and information related to waste production and management have been collected.
c. To verify the validity of EHS RM methodology, results of the Italian case study have been discussed and consistency of conclusions to improve SCP practices has been analysed.

## 3 Materials and methods

### 3.1 EHS RM methodology

To elaborate robust methodology, the ISO 31000 guideline is adopted [64]; in this standard, RM is structured as a proactive decision-making process aiming to prevent and minimize negative future events, by identifying and analysing potential risks, and planning their monitoring and control [116].

As recommended by scientists, multi-criteria evaluation is needed to quantify EHS risks and the main aspects to be included in robust risk assessment methodology are: legal requirements, stakeholders’ expectations, critical issues of production processes, characteristics of operational controls, potential benefits and costs savings (e.g. Refs. [[117], [118], [119], [120], [121], [122]]). Moreover, the RM must be applied to prevent any kind of risk, both in standard operational conditions and in extraordinary and emergency situations. In fact, potential causes of accidents can derive by planned processes but also by infrequent or unplanned events [123,124].

**Fig. 1:**
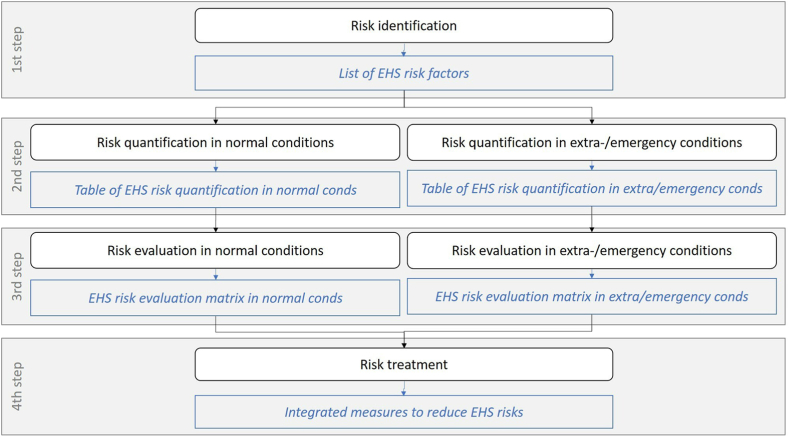
Steps of EHS RM methodology.

EHS RM methodology is designed coherently with the ISO 31000 requirements [64], with the aim to refer simultaneously to EHS risks and to consider both standard conditions and extraordinary/emergency situations. The methodology includes four steps (Fig. 1):

• 1st step – EHS risk identification. Identification of potential hazards is conducted to obtain a single list of EHS risk factors: the expected output is a list of EHS risk factors associated to the waste management.
• 2nd step – EHS risk quantification. Quantification is conducted to assess the EHS risk factors associated with normal conditions and extraordinary/emergency conditions: expected results are risk indexes associated respectively to normal and extraordinary/emergency conditions.
• 3rd step: EHS risk evaluation. Risk evaluation is conducted for each risk index quantified in the 2nd step, to assess its acceptability; two EHS risks evaluation matrices, associated respectively to normal and extraordinary/emergency conditions, are assumed.
• 4th step: EHS risk treatment. Final treatment includes definition of specific measures to reduce the higher risk indexes and safely manage industrial waste under normal and extraordinary/emergency conditions.

### 3.2 Methodology for EHS risk quantification

To assure comprehensive, consistent, and objective assessment, the risk quantification might consider each potential risk associated to process or activity derived by risk identification and estimate the risk index by including several parameters [125].

Through the experience of multidisciplinary team and several qualitative and quantitative methods, a preliminary list of potential risks must be produced, including all the situations in which environmental impacts and/or health&safety accidents can be associated [[126], [127], [128]]. During this step, it is important to include any potential hazard, both in standard and emergency situations, also including possible incorrect behaviours and non-conformity situations.

Preliminary list of potential risks is then subjected to thorough risk quantification. This step includes several criteria, coherently with scientific recommendations (e.g. Refs. [[129], [130], [131], [132]]):

• magnitude of negative effects associated with adverse events;
• probability of occurrence of adverse events;
• control capability to avoid adverse events and/or their negative effects;
• situation/condition considered, as normal (standard operation) or unusual (extraordinary or emergency situation).

On the other hand, risk assessment of industrial waste management can be simultaneously conditioned by other elements related to characteristics of waste, processes, and local conditions [[133], [134], [135]]. Risk index related to industrial waste management activities is elaborated also including the following considerations:

• type of waste, its dangerousness and quantity, due to the characteristics of materials, and substances from which some waste derives, as dimension, severity, persistence, and exposition;
• satisfaction of needs and expectations of interested parties, as legal provisions and concerns of local community, that include requirements to prevent, reduce and control waste production and collection;
• environmental liabilities due to past accidents and fragility of local context due to the presence of other danger's sources;
• actual possibility to prevent accidents and mitigate negative effects through the procedures already in use.

In Table 1 the criteria adopted to quantify the EHS risk factors, associated to normal and extraordinary/emergency conditions, are reported. Ad hoc alternatives, associated with scores, have been supposed for each criterion, based scientific literature experience.

**Table 1:** Criteria and scores to quantify EHS risks factors.

CONDITIONS	CRITERIA (1)	VALUABLE ALTERNATIVES (2)	SCORE (3)
NORMAL	LEGAL (relating to compliance with legal requirements)	Failure to comply with legal limits at least 1 time in 5 years	4
		Suspected non-compliance with legal limits at least 1 time in 5 years	3
		Compliance with legal limits in the past 5 years	2
		Absence of binding legislation	1
	WIDENESS (relating to the vastness of the effects that could occur)	Impact that determines significant economic costs	4
		Impact extended to the local territory/community	3
		Impact contained within the site	2
		Impact contained within the department	1
	TECHNICAL (relating to the technical and organizational solutions already adopted)	Satisfactory control methods cannot be applied	4
		Satisfactory procedures are applied to monitor the impacts	3
		Satisfactory procedures are applied to prevent the impacts	2
		Best available technics are applied to prevent the impacts	1
	PERSISTENCE (relating to the duration of effects)	Persistent effects	4
		Daily effects	3
		Periodical effects	2
		Occasional effects	1
EXTRAOR-DINARY/EMERGENCY	FREQUENCY (relating to the probability of occurrence of the anomalous/emergency situation)	Event happened in the last month	4
		Event happened in the last year	3
		Event happened in the last 5 years	2
		Event never happened in the past 5 years	1
	SEVERITY (relating on the severity of the potential effects)	Impact that determines significant economic costs	4
		Impact extended to the local territory/community	3
		Impact contained within the site	2
		Impact contained within the department	1
	DETECTABILITY (relating to the methods of detection and mitigation)	No detection system	4
		Manual detection and intervention systems	3
		Automatic detection systems and manual intervention systems	2
		Automatic detection and intervention systems	1
	PREVENTION (relating to the prevention methods adopted)	No specific system for prevention	4
		Adoption of procedures and training for emergency response	3
		Adoption of technologies for effects containment	2
		Adoption of technologies for prevention	1

(1) References for criteria: [79,85,88,117,[122], [123], [124],^126^,^130^,^135^].

(2) References for valuable alternatives: [79,80,85,119,121,[128], [129], [130], [131]].

(3) References for score: [55,70,117,118,128,135].

As reported in Table 1, the scores indicate the relevance of risk factors: from score 1 in case of “irrelevant risk factor” to score 4 in case of “extremely relevant risk factor”. For example, when in normal operational conditions the produced waste is subjected to legal requirements (in terms of weight, or chemical/biological characteristics, or disposal restrictions, and so on) and in the past 5 years the company have demonstrated the compliance with these requirements, the score is 2; otherwise, the score is 4 if a failure to comply with legal limits is registered at least 1 time in 5 years. As a further example, with regard to anomalous or emergency conditions related to waste collection or transport or identification, if no critically has occurred in the last 5 years, the score is 1; while if instead a criticality has happened in the last month the score is 4.

Each risk factor has to be assessed by using information collected by managers and from available documentation related to EHS performance of processes. When possible, evidence-based information must be used to select the score for each hazardous situation; the opinion of experts is also essential to obtain a realistic risk evaluation. In case of uncertain data, due to inconsistent or insufficient evidences, a precautional approach is recommended and the reasonably worst case must be considered, then the highest score must be selected [116,125].

The scores assigned to each potential risk are the basis to quantify the risk index.

Equations [F.1] and [F.2] report the formulas for EHS risk index quantification under normal conditions [R(n)] and extraordinary/emergency conditions [R(e)] respectively, including criteria listed in Table 1. Possible scores of R(n) depend on compliance on legal requirements, wideness of effects, risk reduction measures already adopted by company, and persistence of damage. On the other hand, possible scores of R(e) include probability of occurrence of anomalous situations, severity of potential effects, available methods of detection, and prevention measures already adopted by company.

[F.1] R(n)=Legal*Wideness*Technical*Persistence

[F.2] R(e)=Frequency*Severity*Detectability*Prevention

### 3.3 Methodology for the EHS risk evaluation

Risk evaluation assigns an acceptability rating to risk indexes calculated in the previous risk quantification phase; then, it allows to underline unacceptable hazardous situations, in which excessively high risk indexes there are, and urgent actions are needed to reduce them.

**Fig. 2:**
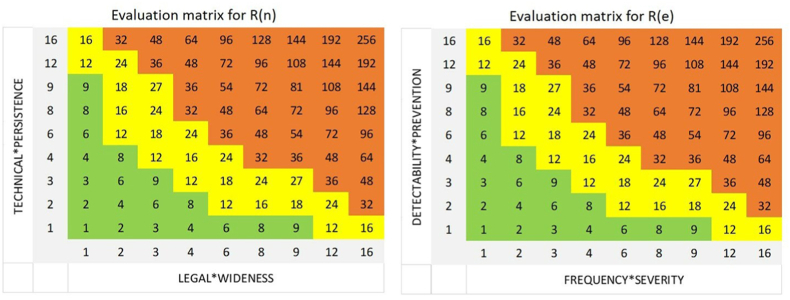
EHS risk evaluation matrix: (Fig. 2 – left) normal conditions [R(n)] and (Fig. 2 – right) extraordinary/emergency conditions [R(e)].

Coherently with international standards recommendations [64,125], ad-hoc evaluation matrixes have to be elaborated to obtain independent and objective conclusions about “negligible risks” with green colour, “critical risks” with yellow colour, and “unacceptable risks” with red colour both under normal and extraordinary/emergency conditions. As represented in Fig. 2, the risk evaluation matrixes are obtained by applying Equations [F.1] or [F.2] respectively in the case of normal or extraordinary/emergency situations:

• in case of risk factors related to normal situations, R(n) evaluation matrix uses two combined parameters: the first parameter is obtained from the multiplication of scores assigned to “legal” and “wideness” criteria, and the second parameter from the multiplication of scores related to “technical” and “persistence” criteria;
• in case of risk factors related to extraordinary/emergency situations, R(e) evaluation matrix considers two combined parameters: the first parameter is the multiplication of scores assigned to “frequency” and “severity” criteria, and the second parameter from the multiplication of scores related to “detectability” and “prevention” criteria.

Lastly, the risk evaluation becomes the base for defining risk treatment procedures. For each EHS risk factor, EHS risk evaluation determines three alternative scenarios:

• if risk index is excessive (red), it must be reduced through urgent treatment actions;
• if risk index is critical (yellow), it can be reduced in a medium term;
• if the risk is negligible (green), no reduction measures are necessary.

### 3.4 Case study

Italian manufacturing enterprise with about 500 employees, world leader in production and distribution of cellulose acetate sheets, is the case study in which the EHS RM methodology was applied. In this company, the main industrial process starts from natural origin polymers to obtain several types of “made in Italy” fashion products, as eyeglasses frames, accessories, and interior design products.

The company has already adopted EHS procedures to minimize the impacts of industrial activities on the workers and the ecosystem, and is interested in further improving internal performances especially in terms of sustainability and circularity, to answer to external stakeholders’ information needs, as customers and commercial partners. At the same time, one of the urgent improvements needed is the integration of EHS procedures to prevent and control accidents.

Within the business strategy, the waste management is a critical issue. Firstly, hazardous waste derived by numerous chemicals used in production processes is one of the main relevant risk factors for both occupational health&safety and environment, already known to consumers and business partners. On the other hand, scraps and residues represent inefficiency to be avoided, primarily for economic reasons.

Testing new risk evaluation model to improve integration of EHS management procedures focusing on the risk minimization in waste collection and disposal is fully in line with the company's strategic objectives. For that, ad-hoc project team has been established, including managers with different skills and experience concerning EHS risk assessment, EHS management system and waste management: business unit heads, plant managers, production managers, warehouse managers, waste management team, and EHS managers have been involved in implementation of EHS RM methodology, to contribute with their own competences and experiences.

## 4 Results and discussion

### 4.1 Results from risk identification

To identify potential risks associated to processes, several audits ware carried out, with the aim of inspecting activities, products, materials, substances, machineries, and plants. Based on observations and interviews of the project team, the main risk factors associated to waste production, collection and transport into operations were identified. Detailed information regarding characteristics of processing waste and potential EHS risks have been collected by interviewing employees, observing production lines and production plants, and warehouses of materials and products, and analysing production data.

To collect adequate and consistent inventory data, related the standard and extraordinary operative conditions, several methods have been adopted. Firstly, meetings with the project team have been organized, and, with a team building approach, the hazardous situations associated to waste management have been listed. Through the experience of the project team members, available evidences supporting planning and control activities have been collected: both technical and human factors have been considered to identify the main potential elements introducing EHS risks in industrial waste management. Secondly quantitative data have been collected by on site audits related to technical characteristics of materials, substances and scraps produced in the industrial plant. Moreover, based on recorded information and the experience of project team members, unsafe and incorrect situations and behaviours occurred in the last three years have been considered.

By using these information and data, preliminary list of risk factors has been finalized by the project team, and for each risk factor consistent quantification has been attributed, in line with the scores in Table 1. Historical datasets of plants, production lines and warehouses have been considered to assign the scores; further insights were made through interviews with the staff dedicated to waste collection/storage/delivery activities. Finally, in case of uncertainty, for example for incomplete or inconsistent data, the worst scores have been selected, to guarantee the precautional approach.

From this preliminary analysis, a detailed list of waste produced in manufacturing and business units have been drawn up: 32 types of waste have been identified, and 13 of which have been classified as dangerous. Table 2 refers to the typology of waste, classified according to the European Waste Catalogue (EWC) using a six-digit code, and an asterisk in case of hazardous material [133].

**Table 2:** Type of waste generated in production activities classified by EWC.

TYPE OF WASTE	EWC CODE	Type of hazard
Sulphuric acid and sulphurous acid	06. 01. 01 *	Absolute Hazardous
Organic halogenated solvents, washing liquids and mother liquors	07. 01. 03 *	Absolute Hazardous
Mineral based non-chlorinated hydraulic oils (not emulsions)	13. 01. 10 *	Absolute Hazardous
Other engine, gear and lubricating oils	13. 02. 08 *	Absolute Hazardous
Engine, gear and lubricating oils	13. 02. 06 *	Absolute Hazardous
Paper and cardboard	15. 01. 01	Absolute Non-Hazardous
Plastic packaging	15. 01. 02	Absolute Non-Hazardous
Wooden	15. 01. 03	Absolute Non-Hazardous
Mixed clean material including packaging	15. 01. 06	Absolute Non-Hazardous
Packaging containing residues of or contaminated by hazardous substances	15. 01. 10 *	Absolute Hazardous
Absorbents, filter materials, wiping cloths, protective clothing	15. 02. 03	Mirror Non-Hazardous
Ferrous metal	16. 01. 17	Absolute Non-Hazardous
Non-ferrous metal	16. 01. 18	Absolute Non-Hazardous
Discarded equipment containing chlorofluorocarbons, HCFC, HFC	16. 02. 11 *	Absolute Hazardous
Discarded equipment containing hazardous components	16. 02. 13 *	Absolute Hazardous
Other discarded equipment	16. 02. 14	Absolute Non-Hazardous
Components removed from discarded equipment	16. 02. 16	Absolute Non-Hazardous
Laboratory chemicals, consisting of or containing hazardous substances	16. 05. 06 *	Mirror Hazardous
Lead batteries	16. 06. 01 *	Absolute Hazardous
Ni–Cd batteries	16. 06. 02 *	Absolute Hazardous
Alkaline batteries	16. 06. 04	Absolute Non-Hazardous
Brick/rubble	17. 01. 02	Mirror Non-Hazardous
Wood	17. 02. 01	Mirror Non-Hazardous
Glass	17. 02. 02	Mirror Non-Hazardous
Bituminous mixtures containing coal tar	17. 03. 01 *	Mirror Hazardous
Copper, bronze, brass	17. 04. 01	Mirror Non-Hazardous
Iron and steel	17. 04. 05	Mirror Non-Hazardous
Mixed metals	17. 04. 07	Mirror Non-Hazardous
Other cables	17. 04. 11	Mirror Non-Hazardous
Other insulation containing hazardous substances	17. 06. 03 *	Mirror Hazardous
Sharps	18. 01. 01	Absolute Non-Hazardous
Fluorescent tubes and other mercury-containing waste	20. 01. 21 *	Absolute Hazardous

Moreover, based on data and information collected and discussed with the project team, preliminary hazard list has been elaborated, in which all the potential hazardous situations have been identified, related to waste production, collection and delivery activities, in normal or extraordinary/emergency conditions.

In normal conditions, EHS risks related to hazardous waste management could be, for example: chemical risk due to the dangerousness of the substances and heat treatments, mechanical risk for moving parts and high temperature surfaces, risk of exposure to intense and prolonged noise, ergonomic risks. In extraordinary and emergency situations, EHS risks could be, for example, mechanical risk for machineries start-up and shutdown, chemical risk related to cleaning and maintenance operations, chemical risks due to disposal waste control failures, chemical risks concerning non-compliance with recycling scraps procedures, fire and explosion risks in case of uncontrolled temperature rise in waste collection areas, soil and wastewater contamination in case of failures in waste collection and transportation.

### 4.2 Results of EHS risk quantification and evaluation

The preliminary hazard list was analysed by the project team on the basis of scores in Table 1: each potential risk factor is quantified considering the relevance of the EHS impacts associated to hazardous situation, that depends on dangerousness of collected waste, classified coherently with the EWC, and dangerousness of processes/machineries/substances, classified coherently with the criteria reported in Table 1.

Equations [F.1] and [F.2] have been applied to all the situations identified in the preliminary hazard list: as R(n) in case of normal conditions, as R(e) in case of extraordinary/emergency conditions. The matrices in Fig. 2 have been applied to identify the risks with unacceptable or critical score. Results obtained by applying the risk quantification and risk evaluation steps have been analysed and discussed with the project team, to verify reliability of conclusions according to the experience of managers.

**Fig. 3:**
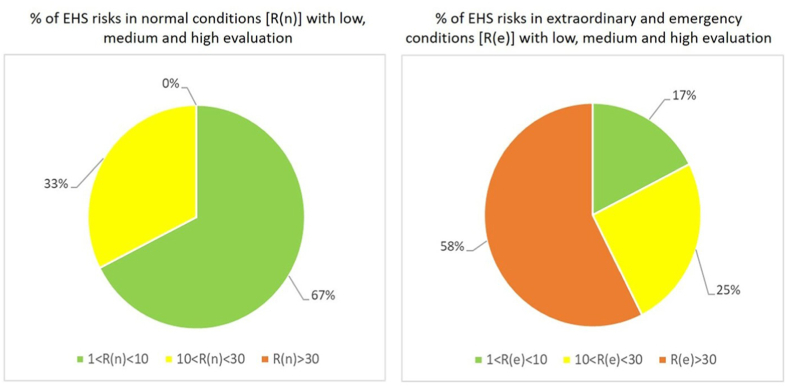
Results of quantification and evaluation of EHS risk: (Fig. 3 – left) in normal conditions [R(n)] and (Fig. 3 – right) in extraordinary/emergency conditions [R(e)].

Fig. 3 summarizes the results obtained by quantification and evaluation of EHS risks in normal conditions [R(n)] and extraordinary/emergency conditions [R(e)].

Considering the case normal conditions (Fig. 3 – left), EHS risks seem substantially under control: more than 60 % of potential risks have been evaluated as “negligible” (green colour), and no treatment measures are necessary. Only 33 % of potential risks identified in normal operations have been evaluated as “critical” (yellow colour), related to specific activities and plants involving handling of hazardous substances and waste, and treatment measures could be implemented to reduce EHS risks associated to these situations.

On the other hand, considering the case of extraordinary and emergency situations (Fig. 3 – right), more than 50 % of identified risks have been evaluated as “excessive” (red colour), with risk index R(e) > 30: in these cases, urgent risk reduction interventions are needed to assure adequate conditions and to avoid inacceptable EHS impacts. However, less than 20 % of risks have been considered as “negligible” with low-risk index (green colour), while 25 % of risks have been evaluated as “critical” (yellow colour) and further improvement measures could be implemented in medium term.

### 4.3 Results of EHS risk treatment

Results obtained from risk identification, quantification, and evaluation have underlined the risks needing more urgent intervention to minimize dangerous conditions, reduce environmental impacts and improve occupational health and safety. As emerged in Fig. 3, risks in extraordinary and emergency conditions represent the main problems for the company. For these risks, immediate treatment actions are necessary to prevent accidents and contain potentially dangerous effects. Moreover, the EHS risk evaluation indicated the most relevant areas of improvement to urgently include in the next planning steps of EHS management system.

The results obtained by the EHS risk assessment have been analysed and discussed with the management team: plant managers, operations managers, HSE managers, and waste managers have been involved in learning the characteristics of hazardous situations, to better understand causes and consequences especially related to the factors with “yellow” or “red” risk indices. For that, in ad-hoc meeting and through risk analysis techniques already in use within the company, each situation with risk index R > 10 has been examined by the project team with failures analysis. The discussion of EHS RM results allowed managers to adopt appropriate measures to reduce EHS risk indices, with targeted corrective actions. By virtue of increased knowledge of EHS risk factors, the project team was able to define appropriate procedures to better control the processes potentially involved in most significant risks, prevent accidents and avoid damages. New EHS management procedures have been defined primarily including preventive measures. For example, detailed instructions have been drawn up to assure correct disposal of waste, layout optimization has been implemented to minimize the leakage of chemicals, additional measures have been adopted to protect workers by the contact with dangerous waste, technical plant improvements have been introduced to intercept principle of fire in waste collection points. New procedures have been also scheduled to intervene as soon as possible in case of accident and solve hazardous situations to minimize damages on health&safety for workers, environment, and community.

By discussing on the results of EHS RM implementation, project team members have been able to gain greater awareness of risks associated with hazardous waste management and, consequently, greater understanding of possible actions to achieve the SCP targets. Risk indices obtained by the EHS evaluation have been used by EHS managers during the internal training courses related the environmental management and health&safety management, to better communicate the exposure risks and raise awareness of all workers about the possible EHS risks. To ensure effective application of new procedures, specific training programs have been scheduled to assure knowledge and understanding of preventive measures, and avoid the most dangerous situations related to production and collection of hazardous waste.

## 5 Conclusions

The research addresses the issue of sustainability and circularity at firm level, and proposes an easy-to-use EHS RM methodology to minimize the industrial hazardous waste. The adoption of SCP is recommended by scientists and international community, but remains difficult to apply by enterprises, because it requires complex competencies often lacked by practitioners. However, a unified RM both for environment and health&safety is undoubtedly convenient.

The case study of Italian manufacturing company world leader in plastic processing represents the concrete situation to design and test ad-hoc EHS RM method to systematically assess and reduce all potential EHS risks associated to industrial waste production and collection, under standard, extraordinary, or emergency conditions. The research experience demonstrates validity of EHS RM method to support the Italian company to assess in an integrated way the environmental potential impacts and the health&safety potential damages: through a unique tool, both environmental and health&safety issues are considered to identify, quantify, evaluate, and treat the main risks associated to hazardous waste production, transportation and collection.

In conclusion, main advantages related to the adoption of EHS RM method for industrial waste minimization are: 1) the opportunity to integrate environmental issues with health&safety considerations through unified risk identification and assessment; 2) the possibility to consider the EHS risks related to both normal conditions and extraordinary/emergency situations; 3) the capability to underline most serious critical issues and threats to be addressed with greater urgency through appropriate corrective actions; 4) the opportunity to gradually implement an integrated RM approach in areas with EHS criticalities - as the waste collection - developing new skills and new solutions to support EHS management systems.

Results obtained by testing EHS RM methodology in industrial waste management confirm the relevance of easy-to-use methods to implement SCP at firm level, and demonstrate the importance of integrated EHS analysis to improve industrial processes both in standard operations and in extraordinary conditions.

The research also demonstrates that in-depth knowledge of production processes and great expertise in identifying risk situations are necessary to implement EHS RM. Concerning the hazardous waste risk assessment, although simplified and standardized criteria can be adopted, detailed information about materials/substances and processes is indispensable, as well as deep awareness of EHS risks is essential. Consequently, multidisciplinary project team, including different competences and experiences, is essential to obtain comprehensive overview of potential risks, and to consider both technical and behavioural elements that contribute to EHS risks. At same time, the EHS RM methodology enriches each member of the project team and improves the consciousness of EHS risks associated to hazardous waste.

EHS RM methodology presented in this paper, although easy to apply, has some limitations related to the criteria used to identify and classify EHS risks, strictly correlated to the risk perception of project team members. When the methodology is to be incorporated into routine process management, it is essential to adopt additional factors, to quantify human factors and other intangible aspects of EHS risks, and reduce the internal uncertainty of risk assessment.

In order to generalize the validity of the research results, some limits have to be overcome with further research. The EHS RM methodology is only tested in a single case study related to waste production and collection. Its applicability must be verified in other industrial processes to check its validity also in relation with other hazardous activities and situations. It is interesting to verify the validity of this EHS RM methodology in other processes in which integrated waste minimization is crucial for SCP targets, for example manufacturing activities in which wide variety of hazardous materials and substances are produced, transported and stocked. Moreover, the methodology could be applied to other industrial sectors, which the SCP targets are crucial to achieve, such as textile, chemicals, energy, and recycling. Other countries have to be included in further tests of EHS RM methodology, in which different legal requirements have to be considered in hazardous waste management, such as other EU and non-EU countries.

Foreseeing future implementations of the EHS RM methodology in other contexts, for example other processes and/or other industrial sectors and/or other countries, it will be necessary at least to (i) re-define the criteria for risk assessment (step 2.) and (ii) re-design the risk matrix in risk evaluation (step 3.), in order to correctly adapt the methodology to specific case studies by including specific technical, normative, and behavioural characteristics.

## Data availability

Data associated with the study will be made available on request.

## Funding

This research did not receive any specific grant from funding agencies in the public, commercial, or not-for-profit sectors.

## CRediT authorship contribution statement

**Anna Mazzi:** Conceptualization, Data curation, Formal analysis, Investigation, Methodology, Resources, Writing – original draft, Writing – review & editing.

## Declaration of competing interest

The authors declare that they have no known competing financial interests or personal relationships that could have appeared to influence the work reported in this paper.
